# Markers to Detect Drinking During Pregnancy

**Published:** 2001

**Authors:** Cynthia F. Bearer

**Affiliations:** Cynthia F. Bearer, M.D., Ph.D., is an associate professor in the Departments of Pediatrics and Neurosciences at Case Western Reserve University, Rainbow Babies and Children’s Hospital, University Hospitals of Cleveland, Cleveland, Ohio

**Keywords:** AODR (alcohol- or other drug-related) biological markers, pregnancy, fetal alcohol effects, birth defects, ethanol metabolism, oxidation, cytochrome P450 2E1, carbohydrate-deficient transferrin, gamma glutamyl transferase, specificity and sensitivity of measurement, medical ethics

## Abstract

Detecting alcohol use among pregnant women is an important step toward preventing alcohol-related birth defects. A biomarker that could detect alcohol use during pregnancy would aid in earlier identification and intervention for affected infants. The existing potential biomarkers for identifying alcohol use during pregnancy can detect varying degrees of alcohol exposure, or use. However, further research is needed to evaluate these biomarkers.

Alcohol use during pregnancy is a significant public health problem. Approximately 14 to 22.5 percent of women report drinking some alcohol during pregnancy (see Stratton et al. 1996). Most women who drink before becoming pregnant either drastically reduce their consumption or completely stop drinking once they are pregnant. One study found that only 4.6 percent of women reported drinking an average of one drink per day by the end of the third trimester of pregnancy, compared with 44 percent before pregnancy (Day et al. 1989). Unfortunately, some women continue to drink heavily during pregnancy. In 1995, 3.5 percent of pregnant women surveyed reported drinking at least five drinks per occasion or at least seven drinks per week (Ebrahim et al. 1998).

Although the proportion of women who drink heavily during pregnancy is relatively small, the total number is large. The costs are high, as heavy drinking during pregnancy can cause fetal alcohol syndrome (FAS), the leading known preventable cause of mental retardation. Drinking during pregnancy can also result in birth defects; neurodevelopmental defects; and subtle deficits on a variety of behavioral, educational, and psychological tests. An estimated 1 percent of all live births are negatively affected by some prenatal alcohol damage, contributing to societal costs estimated at between $75 million and $9.7 billion per year (for caring for patients with mental retardation and surgical repair of associated anomalies).

Identifying alcohol-exposed newborns is difficult; the characteristic facial dysmorphia is most obvious in school-aged children. In one study, researchers missed the diagnosis of FAS in 100 percent of newborns who were diagnosed later in childhood (Little et al. 1990). Early identification of affected infants is desirable because a diagnosis before age 6 is a strong protective factor for secondary problems associated with FAS and fetal alcohol effects (FAE), such as trouble with the law, inappropriate sexual behavior, and alcohol and other drug problems. Therefore, detecting alcohol use during pregnancy is one way to identify infants who will one day be at risk for alcohol-related problems. Currently alcohol use during pregnancy is often underreported. Several short screening tools to detect pregnancy risk drinking have been developed (see pp. 204–209 of this issue for a review of such screening tools; Russell et al. 1994), but no definitive laboratory test is presently available.

Biological markers, or biomarkers, are indicators signaling events in biologic systems or samples (for review, see Subcommittee on Reproductive and Neurodevelopmental Toxicology 1989). Examples of biomarkers include elevated body temperature to signal a fever and blood tests for viral infection. A biomarker that could detect alcohol use during pregnancy might lead to earlier identification of and intervention for affected infants. Such a marker could also help identify women at risk for alcohol use during subsequent pregnancies, help to detect underreporting of alcohol use during pregnancy, and facilitate research on dose-response relationships between alcohol exposure and alcohol-related birth defects (Stratton et al. 1996). The Institute of Medicine’s Committee to Study Fetal Alcohol Syndrome has recommended further research both to develop and to increase the use of biomarkers of alcohol exposure in pregnant women and newborns.

This article will review the development and use of biomarkers in general and focus on biomarkers of maternal drinking during pregnancy.

## Biomarkers

**Figure 1:**
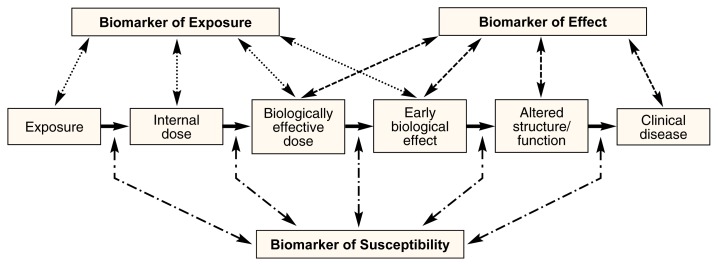
The three categories of biomarkers: biomarkers of exposure, biomarkers of effect, and biomarkers of susceptibility. The markers fall along the spectrum from exposure (e.g., prenatal exposure to alcohol) to disease (e.g., fetal alcohol syndrome). Biomarkers of exposure are designed to detect exposure rather than the effect of exposure. Conversely, biomarkers of effect are designed to detect the effect of exposure or the development of disease. Biomarkers of susceptibility can mark increased vulnerability at any of the steps between exposure and clinical disease.

There are three categories of biomarkers: biomarkers of exposure, biomarkers of effect, and biomarkers of susceptibility. The markers fall along the spectrum from exposure (e.g., prenatal exposure to alcohol) to disease (e.g., FAS) (see figure 1). Biomarkers of exposure are more proximal to the exposure (i.e., they are designed to detect exposure rather than the effect of exposure), and biomarkers of effect are more proximal to clinical disease (i.e., they are designed to detect the effect of exposure, or the development of disease). However, overlap may exist. Biomarkers of susceptibility can mark increased vulnerability at any of the steps between exposure and clinical disease.

**Figure 2:**
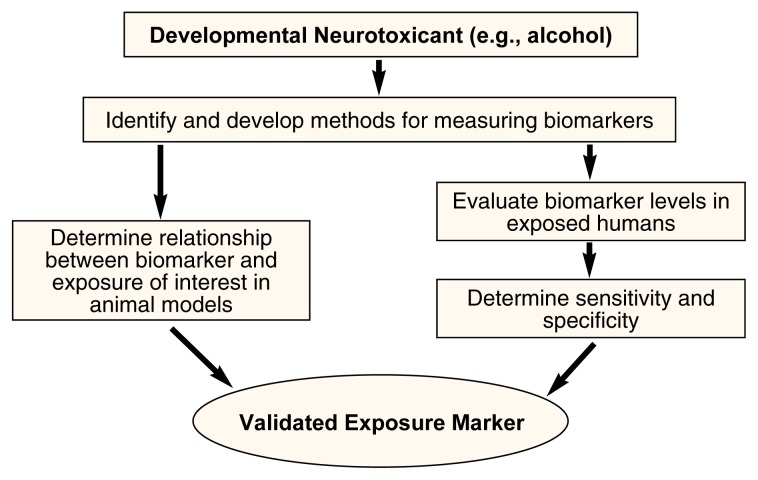
The process of developing a biomarker for alcohol exposure. The first step is to identify a chemical that would indicate alcohol exposure or the effect of exposure and develop methods to measure the biomarker. Validating a biomarker means to ensure that it correctly identifies exposure without false positives or false negatives. Markers of exposure must be validated according to their ability to assess the true exposure (i.e., the sensitivity) and lack of exposure (i.e., the specificity). SOURCE: Adapted from Groopman and Kensler 1999.

Figure 2 shows the process of developing a biomarker of exposure. For this article, alcohol is the suspected developmental neurotoxicant the biomarkers are designed to detect. The first step in developing and validating a biomarker is to identify a chemical that would indicate alcohol exposure or the effect of exposure and that could be detected in a particular biological media (e.g., a product of alcohol metabolism that can be detected in the blood). Table 1 lists some appropriate biomarkers for alcohol exposure as they might fit into the scheme depicted in figure 1. For example, a biomarker that could be used to detect an internal dose of alcohol would be alcohol concentration in the blood. Next, the biological sample (e.g., blood, breath, or urine) used to measure the biomarker must be selected. Practical limitations to obtaining biological samples suitable for biomarker analysis in humans must be considered. First, the samples should involve minimally invasive techniques and test procedures that are readily acceptable to study participants. Also, obtaining the sample should be inexpensive and the sample size (i.e., the amount of the fluid or tissue sample) should be relatively large to increase sensitivity (i.e., the test’s ability to detect exposure). Table 2 lists some appropriate biological samples for both mothers and newborns that could be used to measure biomarkers for maternal alcohol use, along with some advantages and disadvantages for each.

Once a potential biomarker and a potential biological sample have been selected, the next step is to develop a method for analytical quantification of the biomarker in the specific media, or sample. Issues to consider include determination of recovery (i.e., how much of the biomarker will be recovered from the tissue sample, and how much variation there is in recovery of the marker between samples) and the stability of the biomarker in the chosen sample.

The next and most difficult step in developing a biomarker is validating that it correctly identifies exposure without false positives or false negatives (see figure 2). To validate a biomarker, it is necessary to determine the relationship between the biomarker and the exposure of interest. Markers of exposure must be validated according to their ability to assess the true exposure (i.e., sensitivity) and lack of exposure (i.e., specificity). Estimates of sensitivity must consider the background level of the biomarker in a population without exposure as well as the marker’s ability to detect levels of exposure leading to a biological effect. Estimates of specificity also must consider variations in the population, including age and gender, time of day of the measurement, and the effect of other diseases or developmental processes. Ideally, the marker should be specific for the given exposure (i.e., it should not find false positives). When comparing biomarkers, it is necessary to compare their sensitivity and specificity for identifying a similar exposure, or, in this case, a similar level of drinking. Also, because gender and pregnancy also affect biomarkers, biomarkers should be compared within populations of the same sex or pregnancy state.

Validation of a marker also depends on its expected use. Biologic markers observed well before the onset of disease might have little value for predicting the disease, but may be more useful for identifying exposed populations for long-term followup.

Animal models are useful in the validation process because they can be used to study the mechanisms behind the expression of markers and the relationships between markers and exposure. No studies to date have reported on validating an biomarker of drinking during pregnancy using an animal model. We are currently working on a pregnant ewe model for use in further validating a biomarker of maternal alcohol exposure.

## Biomarkers of Maternal Drinking During Pregnancy

Researchers have observed negative outcomes in the offspring of women who consume large amounts of alcohol during pregnancy. Such adverse fetal effects are also tied to moderate drinking but the links are not yet well established. Some investigators have concluded that there is no measurable risk when the mother consumes less than 1 ounce of alcohol per day. However, Streissguth and colleagues (1990) found learning problems in school-age children whose mothers recalled one episode of consuming more than five drinks at once, and Jacobson and colleagues (1994) found deficits on the Fagan Test of Infant Intelligence in 6.5-month-old infants whose mothers consumed on average one drink per day (i.e., seven drinks per week). An ideal biomarker of fetal exposure to alcohol would be sensitive to these levels of alcohol consumption, specific for alcohol consumption, and remain present over time. This article describes several potential biomarkers for prenatal alcohol exposure, focusing on how well they meet these requirements and whether or not they have been validated.

### Marker of Internal Dose

Alcohol concentration, detected on the breath and skin, and in urine, blood, and cord blood (i.e., blood taken from the umbilical cord), is a marker of current use, or internal dose. Although several rapid, sensitive methods for alcohol testing exist (e.g., breath and urine analysis), and the presence of alcohol itself is used as a biomarker for acute alcohol effects (such as violent behavior, or driving while under the influence), alcohol’s rapid elimination from the body makes it insensitive as a biomarker for chronic intermittent alcohol exposure in either pregnant women or newborns.

### Markers of a Biologically Effective Dose

Metabolites of alcohol indicate a level of alcohol consumption associated with biological changes in the body and may be used as markers.

#### Alcohol Metabolism and Products

Alcohol circulating in the blood reaches the liver, placenta, and other metabolic organs of both the mother and the fetus, where it can be broken down or conjugated to other molecules by several enzymes. Enzymes that break down alcohol include alcohol dehydrogenase (ADH) (which oxidizes alcohol to form acetaldehyde) and cytochrome P450 2EI. Enzymes that conjugate alcohol to other molecules include fatty acid ethyl ester (FAEE) synthase, which produces FAEEs, and glucuronyl transferase, which produces ethyl glucuronide. The following products of alcohol metabolism may be useful as biomarkers for alcohol use.

- *Acetaldehyde* can be detected in the blood. Because it is difficult to measure accurately and is quickly eliminated, it is an insensitive marker for chronic intermittent alcohol exposures.
- *FAEEs* are metabolic products that result from the interaction between alcohol and fatty acids. FAEEs can be detected in blood, hair, placenta, cord blood, and meconium (i.e., a waste product of newborns). In one study (Soerberg et al. 1999), FAEEs in serum were detected only up to 24 hours following alcohol ingestion, suggesting that maternal serum FAEEs will not be useful as a biomarker. FAEEs have not been studied in pregnant women. The half-life of FAEEs in mouse placenta is 7 days. The half-life in human placenta, hair, cord blood, and meconium is unknown. FAEEs have been detected in both cord blood and meconium samples from newborns with alcoholic mothers and in meconium samples from infants born to non-alcoholic mothers (Bearer et al. 1999). The possibility of using meconium FAEEs as a biomarker has been explored by researchers. In meconium samples from infants born to nonalcoholic mothers, the presence of one FAEE, ethyl linoleate, was associated with higher weekly levels of alcohol use (based on self-report) during the month prior to pregnancy, in the first trimester, and overall. The biomarker was specific for alcohol. Self-reported use of cocaine, marijuana, and tobacco was similar between mothers whose infants’ meconiums contained the FAEE and those who did not. The sensitivity and specificity of the test were 72 percent and 51 percent, respectively, for distinguishing between women who reported having at least one drink per week in the third trimester and women who denied use. The sensitivity and specificity were 68 percent and 48 percent for distinguishing between women who consumed at least one drink per week and women who consumed less than one drink per week in the month before pregnancy.
  In further studies, researchers found that the quantities of two of the FAEEs, ethyl oleate and ethyl linoleate, correlated with the mothers’ reported drinking in a dose-dependent manner. In addition, FAEE levels in the meconium of infants born to women in an abstaining population were used to establish cutoff values of FAEEs (values correlated with no alcohol exposure). The sensitivity of the test for identifying infants born to mothers who had more than 28 drinks per week in the month prior to pregnancy was 68 percent. The sensitivity was reduced at lower levels of drinking—for more than 14 drinks, more than 7 drinks and more than 3 drinks per week in the month prior to pregnancy, the sensitivity was 63 percent, 45 percent, and 42 percent, respectively, using a cutoff value that gave a specificity of 97 percent in the infants born to abstaining mothers (Bearer et al. 2000*a*, *b*; Bearer et al. 2001). It is likely that FAEEs in meconium may be a useful biomarker for maternal drinking if better analytical tools to measure them could be developed.
- *Ethyl glucuronide,* a minor metabolite of alcohol, is found in adult blood and urine and is detectable in the body for a slightly longer period of time than alcohol. Ethyl glucuronide can be detected in the blood, urine, and hair. It has been measured in adult serum, and has potential as a marker of relapse. No studies have been conducted with pregnant women or newborns.
- *Cocaethylene* is a metabolite of alcohol formed in the presence of cocaine, perhaps by the same enzymes that catalyze the formation of FAEE. It can be detected in the blood, urine, and meconium. It would only be useful as a biomarker in populations with concurrent cocaine use.

### Markers of Early Biological Effect

Both enzymes involved in alcohol metabolism and products created from the interaction of alcohol metabolites and cellular components indicate a level of alcohol consumption consistent with a biological effect on the body and may also serve as markers.

#### Enzymes Involved in Alcohol Metabolism

Some enzymes that are used in alcohol metabolism may serve as biomarkers for alcohol use because they are induced by alcohol, meaning that their concentrations in the body increase in the presence of alcohol.

- *Cytochrome P450 2E1* (CYP450 2E1). This enzyme helps to metabolize alcohol in the liver and may be found at increased levels after chronic drinking. CYP450 2E1 is found throughout the maternal body, including in the liver, brain, and peripheral blood. It is nonspecifically induced by alcohol (i.e., alcohol is one of a number of things that may increase the concentration of the enzyme). Two variants of the enzyme do not appear to differ between alcoholics and control subjects, thus the presence of one variant or another is not a marker for alcoholism. In one report (Lucas et al. 1995), the induction of CYP450 2E1 in alcoholics was found to return to the levels observed in controls, after the alcoholics abstained for 8 days. Researchers have estimated the half-life of the protein at 2.5 days. In a rabbit model, P450 2E1 levels could be induced 6- to 10-fold when the animals were given 15 percent alcohol in their drinking water for 12 days. The researchers found that the extent of the induction correlated well with blood alcohol concentration (BAC) and could be demonstrated at BACs as low as 50 mg/dL^1^ (Raucy et al. 1995). However, in human studies, researchers found only a 2.3-fold increase in P450 2E1 levels between alcohol abusers and controls (Raucy et al. 1997). No studies of pregnant women or newborns have been reported.
- *Catalase* is also an enzyme induced by alcohol and detected in the blood. One study (Koechling and Amit 1992) of Caucasian volunteers found a positive relationship between self-reported alcohol consumption and catalase activity in peripheral blood. No further work has been reported on developing blood catalase as a biomarker of maternal alcohol consumption or fetal alcohol exposure.
- *FAEE synthase,* an enzyme involved in alcohol metabolism, has been found to be active in most organs of the body and in peripheral blood, cord blood, placenta, and meconium. Several reports have shown that tissue-specific FAEE synthase activity can be altered following chronic alcohol exposure. For example, the FAEE synthase activity in white blood cells taken from control subjects was twice that observed in alcoholics admitted to a detoxification unit. Another study showed that FAEE activity in white blood cells of healthy non-alcoholic volunteers could be induced nearly 2-fold upon ingestion of 2 ounces of Scotch whiskey per day for 6 days, whereupon the activity returned to control levels despite continued ingestion of 2 ounces of Scotch whiskey for an additional 3 days (Gorski et al. 1996). These results suggest that FAEE synthase activity may be a useful marker of alcohol use for binge drinkers, although the dynamics of enzyme expression appear to be complex and the changes described occur at high alcohol doses. FAEE synthase activity has not been studied in pregnant women or newborns.

#### Products of the Interaction of Alcohol Metabolites and Cellular Components

The following potential biomarkers are formed when products of alcohol metabolism interact with other cellular components in the body to create compounds that can be detected in the blood or urine.

- *Acetaldehyde-protein adducts.* Because acetaldehyde is rapidly metabolized it does not accumulate in the body and therefore has limited potential as a biomarker. However, acetaldehyde can form adducts (i.e., compounds) with various proteins during chronic alcohol exposure, and these adducts can be detected in the blood. Proteins with detectable acetaldehyde adducts include hemoglobin, serum proteins, albumin, CYP450 2E1, red blood cell membrane proteins, and a number of other enzymes. One study (Sillanaukee et al. 1992) found that the concentration of hemoglobin-acetaldehyde (Hb-Ac) was significantly higher in 18 heavy drinkers and 20 alcoholics compared with 22 control subjects. In addition, the sensitivity to determine heavy drinking was 50 percent, higher than the sensitivity for two of the traditional biomarkers of alcohol abuse, gamma glutamyltransferase (GGT) (39 percent sensitivity) and mean corpuscular volume (17 percent sensitivity). In another study (Hazelett et al. 1998), when a cutoff value of Hb-Ac chosen to maximize both sensitivity and specificity was used to detect drinking more than six drinks per day versus less than six drinks per day, the sensitivity and specificity were 67 percent and 77 percent respectively. At a specificity of 100 percent, sensitivity dropped to 20 percent. Researchers found that one dose of alcohol significantly increased the concentration of Hb-Ac in control subjects, indicating that chronic versus acute exposure cannot be determined by this test. One study (Niemala et al. 1991) tracked the Hb-Ac adducts for 19 pregnant problem drinkers. Four women became abstinent during the pregnancy and 15 continued to drink 10 to 35 drinks per week. Researchers examined the newborns for characteristics of FAE. The Hb-Ac values were elevated in five of eight of the women who gave birth to children with FAE, compared with the values for pregnant women who abstained from alcohol, and the values were elevated in two of the seven women who, despite drinking, delivered healthy children. Thus the test would identify approximately 50 percent of pregnant alcohol abusers. Further studies may be useful in determining the potential of this biomarker.
- *Oxidation products.* Alcohol metabolism involves a number of processes, one of which is oxidation. Through oxidation, alcohol is detoxified and removed from the blood, preventing the alcohol from accumulating and destroying cells and organs. Oxygen radicals produced during oxidation can then react with lipids to form lipid peroxidation products, compounds such as dienes and malondialdehyde, which may act as biomarkers detectable in the blood. Baldi and colleagues (1993) used serum malondialdehyde levels to distinguish between 15 healthy control subjects and 3 groups of alcoholics— those with normal liver function, those with non-cirrhotic alcoholic liver disease, and those with cirrhotic alcoholic liver disease. The researchers found that, irrespective of the presence of liver disease, using malondialdehyde as a biomarker for alcohol use had a sensitivity of 70 percent and a specificity of 100 percent. In another study (Butcher et al. 1993) the concentration of dienes was higher in an alcohol-using population compared with normal controls, suggesting the potential for this biomarker for alcohol use, which could be detected in either blood taken from pregnant women or in cord blood.
- *5-Hydroxytryptophol/5-Hydroxyindole-3-acetic acid (5–HTOL/5–HIAA).* Five-hydroxyindoleacetic acid (5-HIAA) is produced when the brain chemical serotonin is broken down. At the same time, the alcohol metabolite 5-hydroxytryptophol (5–HTOL) is formed. Under normal circumstances and without the intake of alcohol, the ratio of 5–HTOL/5–HIAA is small. When the body is engaged in alcohol metabolism, the ratio of 5–HTOL/5–HIAA increases in blood and urine. Researchers have reported elevated ratios of 5–HTOL/5–HIAA in men and women attending a methadone clinic who reported recent drinking (Helander et al. 1999). The mean alcohol intake of those with elevated ratios was 60 grams or approximately 5 drinks per day. This ratio was significantly increased in preoperative chronic alcoholics. Researchers have not reported either the sensitivity or specificity of this test, and no studies have been conducted in pregnant women or newborns.

### Altered Structure/Function

Markers indicating levels of alcohol use high enough to result in alterations in normal body structures or functions include altered target proteins and early indications of target organ damage.

#### Alteration of Target Protein

The following markers are proteins that increase in concentration in response to alcohol consumption.

- *Carbohydrate-Deficient Transferrin (CDT),* a blood protein that increases in concentration after alcohol consumption, has recently received much attention for its potential as a biomarker for alcohol exposure. One review of 16 studies that examined CDT as a biomarker in women with alcohol problems (Allen et al. 2000) reported that the median sensitivity was 51 percent for all studies distinguishing different degrees of severity of drinking over a range of drinking behaviors from women drinking less than 2 drinks per day, with a median specificity of 92 percent. However, the sensitivity falls dramatically when comparing heavy drinkers with moderate drinkers. The promise of this biomarker increases when used in combination with other tests. CDT concentrations in cord blood are significantly higher than in maternal blood, and are not correlated with reported maternal alcohol intake.
- *Serum proteins* may be useful for analysis in detecting FAS in children. In one study, researchers analyzed the serum proteins of 12 FAS patients and 8 age- and sex-matched control subjects and found 8 proteins with significant concentration differences between the FAS patients and the control subjects (Robinson et al. 1995). No single protein differentiated all FAS patients from the control subjects, but a panel of four proteins did. It is unclear how this test will perform in identifying infants or children with alcohol-related birth defects who do not have clear signs of FAS. The research also did not determine if the same panel of four proteins could distinguish the mothers of the affected children from the control mothers.
- *Urinary dolichols* are chemicals found in high levels in urine excreted by alcoholics. One study tracked the urinary dolichols among 16 infants who were small for gestational age (Wisniewski et al. 1983). Among 6 of the 16 infants born to mothers who were heavy drinkers, 2 had FAS and 4 had effects consistent with alcohol-related birth defects. All 6 had 4 to 5 times higher urinary dolichols levels (20–38 ng/mL) compared with infants without prenatal exposure to alcohol (2–7 ng/mL). No followup or other study has been published on this potential marker. However, the fact that there was no overlap in dolichol levels between exposed and unexposed infants makes this biomarker intriguing and possibly very useful. Dolichols have not been studied in meconium.
- *Sialic Acid,* a chemical detected in the blood, is found in increased concentrations in alcoholics. One study that measured sialic acid levels in social drinkers and alcoholics reported that the sensitivity and specificity of this marker to distinguish these two groups was 57.7 and 95.5 for women, and 47.8 and 81.3 for men (Sillanaukee et al. 1999). This marker needs further evaluation in pregnant women.

#### Early Indication of Target Organ Damage

This section reviews some conventional biomarkers that are used to detect heavy alcohol use. These markers detect cellular changes that reflect early signs of organ damage in response to heavy drinking.

- *Gamma glutamyltransferase.* Elevated blood levels of this enzyme indicate long-term heavy alcohol use. In 10 studies of women with alcohol problems, the median sensitivity of GGT for detecting women diagnosed with alcohol problems was 54 percent, with a median specificity of 96 percent (Allen et al. 2000). Thus CDT and GGT have similar sensitivities and specificities in women.
- *Aspartate aminotransferase (AST)/alanine aminotransferase (ALT).* These liver enzymes, which can be detected in the blood, can be useful markers for alcohol abuse. One study found that AST and ALT were comparable in sensitivity and specificity to distinguish social drinkers from alcoholics (Sillanaukee et al. 1999). The sensitivity and specificity of AST were 53.8 and 95.5 percent for women and 43.5 and 100.0 percent for men. ALT had a sensitivity and specificity of 38.5 and 90.9 percent for women and 39.1 and 87.5 percent for men, respectively.
- *Mean corpuscular volume (MCV),* an index of red blood cell size, increases with excessive alcohol intake. Mundle and colleagues (2000) compared the predictive value of MCV, CDT, and GGT in men and women. In women, MCV was found to be superior, with a sensitivity of 86 percent and a specificity of 90 percent, compared with CDT (49 and 90 percent) and GGT (60 and 90 percent).
- *Beta-hexosaminidase.* Tests for the enzyme beta-hexosaminidase, which can be detected in the blood, have been used among males to distinguish alcoholics from moderate drinkers and abstainers. The test had a sensitivity of 94 percent and a specificity of 91 percent in detecting alcohol consumption of more than 60 grams per day. This biomarker should be further investigated in women and pregnant women, perhaps as part of a battery (Stowell et al. 1997).

## Test Batteries

Researchers have also tested combinations of biomarkers in attempts to improve sensitivity and specificity. For one study, researchers developed an “alcohol index” using four markers, including GGT and CDT. Using this index, the authors reported achieving 100 percent specificity and 93 percent sensitivity in distinguishing alcoholics from three groups of non-alcoholics (Brinkmann et al. 2000). Harasymiw and colleagues (2000) used an algorithm of 34 blood chemistry measurements to calculate an Early Detection of Alcohol Consumption (EDAC) score. Based on this score, they demonstrated a 100 percent sensitivity and 82 percent specificity in identifying women who drank at least three drinks per day, and 42 percent sensitivity and 90 percent specificity when identifying women who drank at least seven drinks per week or more than three drinks on any occasion. In 5 studies using both CDT and GGT in women with alcohol problems, the median sensitivity in detecting alcoholics entering treatment or heavy drinkers (i.e., those consuming more than 140 grams per week) was 44 percent for each test separately and 72 percent when used in combination, with a specificity of 90 percent (Allen et al. 2000). In one study of pregnant women with alcohol abuse, hemoglobin-acetaldehyde adducts and CDT were not associated with the reported level of drinking (Sarkola et al. 2000). However, MCV and GGT were significantly higher in women drinking at least eight drinks per week compared with those drinking less than eight drinks per week. Specificity and sensitivity were not reported. In another study among pregnant women, tests for CDT, GGT, MCV, and hemoglobin-acetaldehyde adducts were combined (Stoler et al. 1998). All women who reported drinking at least 14 drinks per week were positive for 1 or more markers. Having two or more positive markers was more predictive of infant outcome than any measure of self-reported drinking. However, sensitivity and specificity were not reported in this study.

## Ethical Considerations

A number of ethical issues complicate the use of biomarkers for detecting alcohol use among pregnant women. Informed consent is sometimes needed to analyze discarded samples such as cord blood, placenta, or meconium for alcohol and other drugs. This policy varies and is highly individualized to the hospital where such specimens are collected. Regulations governing how physicians must respond when indicators identify maternal drug or alcohol use also vary by state. Some states currently require physicians to report women who test positive for alcohol or other drug use to local departments of health and human services. Such women do not need to be punished, but should be directed toward treatment programs.

## Conclusion

Detecting alcohol use among pregnant women is an important step toward preventing alcohol-related birth defects. In addition, the early identification of exposed infants may lead to new modalities of therapy. Thus, biomarkers of maternal alcohol use should be developed and used for primary or secondary prevention. Though several potentially useful biomarkers of maternal alcohol use during pregnancy are being developed and tested, much research is still needed to validate their use.

## Figures and Tables

**Table 1:** Biomarkers of Maternal Drinking

Level of Exposure Indicated by Biomarker	Type of Biomarker	Examples
Internal dose	Alcohol concentration	Blood alcohol concentration
Biologically effective dose	Metabolites of alcohol	Acetaldehyde
		Fatty acid ethyl esters (FAEEs)
		Ethyl glucuronide
		Cocaethylene
Early biological effects	Enzymes involved in alcohol metabolism	Cytochrome P450 2E1
		Catalase
		FAEE synthase
	Products of the interaction of alcohol metabolites and cellular components	Acetaldehyde-protein adducts
		Oxidation products
		5-hydroxytryptophol/5-hydroxyindole-3-acetic acid (5–HTOL/5–HIAA)
Altered structure/function	Alteration of target protein	Carbohydrate-deficient transferrin
		Serum proteins
		Urinary dolichols
		Sialic acid
	Early indication of target organ damage	Gamma glutamyltransferase
		Aspartate aminotransferase/alanine aminotransferase
		Mean corpuscular volume
		β-hexosaminidase

**Table 2:** Biological Samples for Measuring Biomarkers Indicating Maternal Alcohol Consumption

Biological Sample	Advantages	Disadvantages
**Maternal Sample**		
Urine	Large sample size	Requires cooperation; tampering possible
Hair	May indicate timing of exposure	Requires cooperation; may not be desirable; requires special analytical techniques
Blood	Battery of biomarkers may be used	Invasive; painful
Breath	Easy to obtain large quantities	Requires special equipment; technology is limited; requires cooperation
Saliva	Easy to obtain	Requires cooperation; small sample size
Transdermal	Easy to obtain	Requires special equipment; technology is limited; requires cooperation
Newborn Sample*		
Cord blood	Large sample size; battery of biomarkers may be used	Narrow window of opportunity to collect; single time point for measurement
Placenta	Large sample size	Narrow window of opportunity to collect
Umbilical cord	Large sample size	Narrow window of opportunity to collect
Amniotic fluid	Large sample size	Difficult to collect; narrow window of opportunity to collect
Urine	Concentrates metabolites	Difficult to collect
Hair	May indicate timing of exposure	May not be available; may not be acceptable to parent
Breath	Easy to obtain	Requires special equipment; technology is limited
Saliva	Easy to obtain	Small sample size
Transdermal	Easy to obtain	Requires special equipment; technology is limited
Meconium	Easy to obtain; may indicate timing of exposure	None

* Biomarkers measured in newborn samples only indicate maternal drinking retrospectively.

## Footnotes

1 BAC is the proportion of alcohol to blood in the body. BAC is expressed as milligrams of alcohol per deciliters (dL) of blood.
